# Design and Synthesis of a High-Performance Copper(II) Metal–Organic Framework Featuring Large Surface Area and Enhanced Methane Adsorption Capacity from a Thiophene-Functionalized Diisophthalic Acid Ligand

**DOI:** 10.1021/acs.inorgchem.6c00691

**Published:** 2026-03-30

**Authors:** Okan Zafer Yeşilel, Ferihan Tataş Coşkun, Ahmet Safa Aydogdu, Alper Uzun, Hasan Can Gulbalkan, Seda Keskin, Kamuran Görgün, Mürsel Arıcı

**Affiliations:** † Department of Chemistry, Faculty of Science, 372380 Eskişehir Osmangazi University, 26040 Eskişehir, Turkey; ‡ Department of Chemical and Biological Engineering, 52979 Koç University, Rumelifeneri Yolu, 34450 Sariyer, Istanbul, Turkey; § Koç University Hydrogen Technologies Center (KUHyTech), Koç University, Rumelifeneri Yolu, 34450 Sariyer, Istanbul, Turkey; ∥ Koç University Surface Science and Technology Center (KUYTAM), 52979 Koç University, Rumelifeneri Yolu, 34450 Sariyer, Istanbul, Turkey

## Abstract

In this study, a new copper-based metal–organic framework, {[Cu_3_(μ_8_-mtif)_1.5_(DMF)_3_]·10H_2_O·4DMF}_*n*_ (**OGU-3**), was synthesized using 5,5′-(3-methylthiophene-2,5-diyl)­diisophthalic acid (H_4_mtif) as an organic linker through a solvothermal method. Single-crystal X-ray diffraction analysis revealed that the **OGU-3** possesses a three-dimensional porous architecture with a high surface area (2612 m^2^/g). The framework contains one-dimensional channels along the *c-*axis with dimensions of approximately 11.26 × 15.48 Å. Gas adsorption measurements demonstrated that the material exhibits a remarkable CH_4_ uptake capacity, attributed to its large surface area and optimized pore environment. High-pressure CH_4_ adsorption measurements revealed that **OGU-3** exhibits a remarkable gravimetric CH_4_ uptake of 292.5 cc (STP)/g at 25 °C and 65 bar, representing 5.4% enhancement over its nonmethylated analogue. The findings highlight the potential of thiophene-functionalized ligands in designing high-performance MOFs for efficient CH_4_ storage applications. This work provides valuable insights into the development of advanced porous materials for energy-related gas storage technologies.

## Graphical abstract

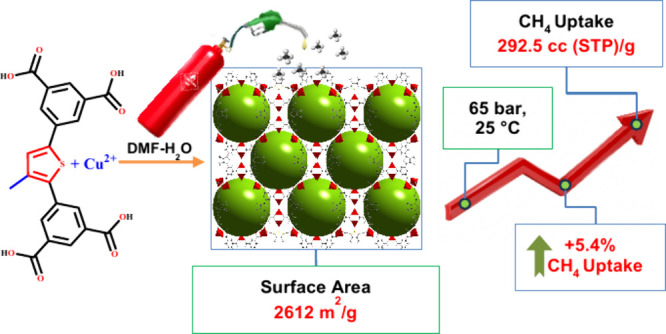

## 1 Introduction

Metal–organic frameworks (MOFs) have emerged as a versatile class of crystalline porous materials constructed from metal nodes and organic linkers.^1^ Their exceptional surface areas, tunable pore structures, and chemical functionalities make them attractive candidates for a wide range of applications, including gas storage, separation, catalysis, and sensing. Among these, the storage of methane (CH_4_), a promising alternative fuel with high energy density and cleaner combustion properties, has been a critical target owing to the growing demand for efficient and safe storage systems.^2,3^

Copper-based MOFs (Cu-MOFs) represent one of the most extensively studied families of MOFs for CH_4_ storage, thanks to their robust frameworks and favorable pore environments. One of the benchmark materials, HKUST-1 (Cu_3_(BTC)_2_ where BTC = 1,3,5-benzenetricarboxylate), has been widely investigated and exhibits a high CH_4_ uptake capacity (∼230 cc (STP)/cc at 298 K and 35 bar).^4^ Nevertheless, despite their stability and accessibility, many Cu-MOFs face limitations in CH_4_ storage under practical conditions, largely due to factors such as suboptimal pore sizes, limited interaction sites, and framework flexibility. Therefore, the design and synthesis of new Cu-MOFs with improved surface areas and enhanced adsorption sites remain highly desirable for advancing CH_4_ storage technologies.^5,6^

Recent efforts have focused on introducing heteroatoms (such as sulfur, nitrogen, and oxygen) into organic linkers to enhance framework-gas interactions and increase gas uptake capacities.^7,8^ Thiophene-functionalized linkers, containing sulfur atoms, are particularly promising because they offer additional polarizable sites that can strengthen interactions with gas molecules. However, MOFs constructed from thiophene-derived diisophthalic acid linkers remain relatively underexplored, and their methane adsorption properties have not been systematically studied.

In this work, we report the design and synthesis of a new Cu-based MOF, {[Cu_3_(μ_8_-mtif)_1.5_(DMF)_3_]·10H_2_O·4DMF}_*n*_ (**OGU-3,** OGU for Osmangazi University), assembled from a thiophene-functionalized tetracarboxylic acid linker, 5,5′-(3-methylthiophene-2,5-diyl)­diisophthalic acid (H_4_mtif). The structure of H_4_mtif was determined by ^1^H-Nuclear Magnetic Resonance (NMR) spectroscopy. The **OGU-3** was characterized using Fourier Transform Infrared (FT-IR) spectroscopy, elemental analysis, and single-crystal X-ray diffraction (SC-XRD) techniques. The phase purity and thermal stability of **OGU-3** were investigated using powder X-ray diffraction (PXRD) and thermogravimetric analysis (TG/DTA), respectively. The resulting framework displays a three-dimensional porous structure with a Brunauer–Emmett–Teller (BET) surface area exceeding 2600 m^2^/g. Experimental gas adsorption measurements and molecular simulations reveal that **OGU-3** exhibits a high CH_4_ storage capacity, surpassing that of most conventional Cu-based MOFs under similar conditions. The combination of high surface area and the presence of sulfur functionalities is proposed to contribute synergistically to the observed CH_4_ uptake. This study provides valuable insights into the role of heteroatom-functionalized ligands in the development of advanced MOFs for efficient CH_4_ storage.

## 2 Experimental Methodology

### 2.1 Materials and Measurements

All chemicals were purchased from commercial sources. The FT-IR spectra were recorded over the range 4000–400 cm^–1^ on a Bruker Tensor FT-IR spectrometer with the use of KBr pellet. Elemental analyses of the compounds for C, H, N, and S were performed with a PerkinElmer 2400 Series II device. Thermogravimetric analyses (TG, DTA and DTG) were performed on a PerkinElmer Diamond TG/DTA Thermal Analyzer under a dry air atmosphere with a temperature range from 303 to 1273 °C. ^1^H and ^13^C NMR spectra were recorded in DMSO-*d*_6_ using the residual solvent signal as an internal standard on a Jeol ECZ 500R (500 MHz) spectrometer at room temperature. A Panalytical Emperian X-ray diffractometer with Cu-Kα radiation was used to record powder X-ray diffraction patterns. Variable-temperature PXRD patterns of **OGU-3** were collected on a Panalytical Empyrean diffractometer equipped with an Anton Paar HTK 1200 furnace, in air over the temperature range of 298–573 K, with a heating rate of 5 K/min. Gas adsorption measurements were performed with Micromeritics TriStar II Plus.

A Bruker Smart Apex II CCD diffractometer outfitted with a Mo-Kα radiation (λ = 0.71073 Å) was used for single crystal X-ray diffraction data collection. The crystal structures were determined using the SHELXT-2015 program^9^ in conjunction with OLEX2 ^10^ employing the intrinsic phasing method. Refinement of all non-hydrogen atoms was carried out anisotropically using the full-matrix least-squares method on F^2^ with SHELXL-2015,^11^ while hydrogen atoms were placed in geometrically calculated positions. The coordinated and solvent molecules exhibited severe disorder and could not be satisfactorily modeled. Consequently, the solvent masking procedure implemented in OLEX2 was applied to account for the diffraction contributions of the disordered solvent regions. The solvent mask analysis revealed 2753 electrons within a volume of 11,481 Å^3^ corresponding to one void per unit cell. This finding is consistent with the presence of six coordinated DMF molecules, 20 water molecules, and eight uncoordinated DMF molecules per asymmetric unit, accounting for approximately 2848 electrons per unit cell (*Z* = 4). TG and elemental analysis results also supported the solvent masking calculation. The crystal structures were drawn using the Mercury program.^12^ ToposPro software was used for topological analysis of the compounds.^13^

Volumetric CH_4_ adsorption measurements were conducted using a Quantachrome Instruments High-Pressure Volumetric Gas Adsorption Analyzer (iSorb HP2). Prior to measurements, approximately 0.2 g of the sample was degassed under vacuum at 353 K for 3 h. Following the degassing, CH_4_ adsorption isotherms were collected up to 65 bar. The experimental isotherms were fitted using the Dual-Site Langmuir (DSL) model implemented in the IAST++ software.^14^ The isosteric heat of adsorption (*Q*_st_) was calculated using the Clausius–Clapeyron equation from CH_4_ adsorption isotherms measured at 298, 303, and 308 K over a pressure range up to 65 bar.

### 2.2 Synthesis of the Compounds

#### 2.2.1 Synthesis of 5,5′-(3-Methylthiophen-2,5-diyl)­diisophthalic Acid (H_4_mtif)

The synthetic route to 5,5′-(3-methylthiophen-2,5-diyl)­diisophthalic acid (H_4_mtif) is outlined in Scheme S1. 2,5-Dibromo-3-methylthiophene (I) (0.799 g, 3.12 mmol) was dissolved in anhydrous tetrahydrofuran (THF, 20 mL), followed by the addition of dimethyl 5-(4,4,5,5-tetramethyl-1,3,2-dioxaborolan-2-yl)­isophthalate (II) (2 g, 6.24 mmol) and tetrakis­(triphenylphosphine)­palladium(0) (Pd­(PPh_3_)_4_) (0.083 g, 0.07 mmol). Subsequently, deionized water (20 mL) was added, and the mixture was treated with potassium carbonate (K_2_CO_3_, 1.04 g, 7.50 mmol). The reaction mixture was degassed under a nitrogen atmosphere for 15 min and then refluxed for 12 h under nitrogen using a reflux condenser. Upon completion, the reaction mixture was concentrated under reduced pressure using a rotary evaporator, and the residue was extracted with dichloromethane (CH_2_Cl_2_). The organic layer was dried and concentrated, and the crude product was purified by column chromatography (1:1, CH_2_Cl_2_: *n*-hexane) to afford tetramethyl 5,5′-(3-methylthiophene-2,5-diyl)­diisophthalate (III) as a solid (1.20 g, 91% yield).

A mixture of compound III (1.20 g, 2.87 mmol) and KOH (1.50 g, 26.7 mmol) was refluxed in a solvent system consisting of THF (15 mL) and distilled water (10 mL) for 24 h. Upon completion of the reaction, the solvent was removed under reduced pressure. The resulting residue was washed with dichloromethane (CH_2_Cl_2_), filtered, and subsequently acidified with 6N hydrochloric acid. The precipitated solid was collected by filtration, thoroughly washed with water, and dried under vacuum to afford H_4_mtif as a solid product (1.35 g, 83% yield) (Scheme S1). The molecular structure of the tetracarboxylic ligand H_4_mtif is shown in Scheme 1. ^1^H NMR (500 MHz, DMSO-*d*_6_): δ 13.47 (s, 4H, −OH), 8.40 (t, *J* = 1.6 Hz, 1H, H4′), 8.33 (s, 3H, H2, H4, H6), 8.22 (d, *J* = 1.6 Hz, 2H, H2′, H6′), 7.67 (s, 1H, H4), 2.33 (s, 3H, CH_3_) (Figure S1), ^13^C NMR (125 MHz, DMSO-*d*_6_) δ 166.76, 139.97, 135.86, 134.93, 133.00, 132.89, 132.65, 130.46, 129.79, 129.38, 15.55 (Figure S2).

**Scheme 1:**
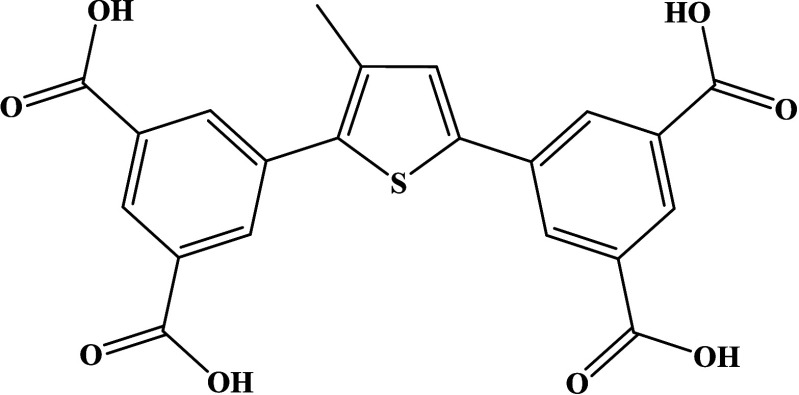
Structure of 5,5′-(3-Methylthiophen-2,5-diyl)­diisophthalic Acid (H_4_mtif)

#### 2.2.2 Synthesis of OGU-3

A mixture of Cu­(NO_3_)_2_·3H_2_O (0.3 mmol, 72.3 mg) and H_4_mtif (0.1 mmol, 42.6 mg) was dissolved in a mixture of 5 mL of DMF and 3 mL of water. Then, 6 drops of concentrated HNO_3_ were added to the mixture and sonicated at 298 K for 30 min. The reaction mixture was taken in a 25 mL closed capped bottle and heated at 348 K for 1 day. The blue crystals were formed, washed with a DMF/water mixture, and dried in air. Yield: 25.07 mg (85.0%). FT-IR (ν, cm^–1^): 3423, 2992, 1662, 1591,1438, 1375 (Figure S3). Anal. Calcd for C_105_H_168_Cu_6_N_14_O_58_S_3_: C, 40.93; H, 5.56; N, 6.55%. Found: C, 41.59; H, 5.58; N, 6.47%. **ZJNU-117** ({[Cu_2_L_2_(H_2_O)_2_]·6DMF}_*n*_) was synthesized according to a previously reported method.^8^

### 2.3 Computational Methodology

Molecular simulations were performed to compute the CO_2_ and CH_4_ uptakes of **OGU-3** and **ZJNU-117**. Some modifications were made to the crystallographic information file (CIF) of **OGU-3** before performing simulations: one of the methyl groups that appeared as a split position as a result of PXRD measurements was removed to maintain structural consistency. Additionally, the oxygen atoms of the solvent molecules coordinated to the metal nodes were removed, as they were lost during the activation process under experimental conditions, resulting in open Cu­(II) sites. Grand Canonical Monte Carlo (GCMC) simulations were carried out using the RASPA simulation package^15^ (version 2.0.5) to obtain the single-component adsorption of CO_2_ and CH_4_ in **OGU-3** and **ZJNU-117** over the pressure range of 0.01–1 bar at 273 and 298 K. CO_2_ was modeled using the TraPPE force field as a three-site rigid molecule with partial charges assigned to each interaction center.^16^ CH_4_ was modeled as a single-site spherical molecule.^17^ Dispersion interactions between MOFs and gas molecules were described using Lennard-Jones 12-6 (LJ) potential. The Universal Force Field (UFF) was employed to model the van der Waals interactions of the framework atoms.^18^ PACMAN (Partial Atomic Charge Predictor for Nanoporous Materials) charges,^19^ which provide the density functional theory (DFT)-quality charges consistent with those derived from the density derived electrostatic and chemical (DDEC) method, were assigned to the framework atoms to calculate electrostatic interactions between CO_2_ molecules and MOF structures. All short-range interactions were truncated at a cutoff distance of 13.0 Å. Long-range electrostatic interactions were considered using the Ewald summation.^20^ Each GCMC simulation consisted of 30,000 cycles, including 10,000 equilibration cycles followed by 20,000 production cycles for ensemble averaging.

## 3 Results and Discussion

### 3.1 Crystal Structure

Crystal data and structure refinement parameters for the **OGU-3** are presented in Table S1. Selected bond lengths and angles are listed in Table S2, provided in the Supporting Information (SI). Single-crystal X-ray diffraction analysis reveals that **OGU-3** crystallizes in the orthorhombic crystal system in the space group of *Cmcm*. The asymmetric unit of **OGU-3** contains one and a half Cu­(II) ions, one-half of a mtif ligand, one-quarter of another mtif ligand (Figure 1). The coordinated and uncoordinated solvent molecules were highly disordered and could not be modeled. Hence, they were removed from the structure using a solvent masking protocol. Two neighboring Cu­(II) ions are bridged by four bis­(monodentate) carboxylate groups of the four different mtif ligands to form a paddlewheel [Cu_2_(COO)_4_] secondary building unit (SBU), where the neighboring Cu­(II) ions are separated by 3.0850(4) Å. These SBUs are further connected through the mtif linkers to construct a three-dimensional porous MOF. As shown in Figure 1, the Cu­(II) ions are five*-*coordinated, locating in a nearly ideal square pyramidal coordination environment (τ = 0 for Cu1 and Cu2 and 0.02 for Cu3) in [Cu_2_(COO)_4_] (Tau (τ) = (β – α)/60, where α and β are the longest and second longest angles, respectively. τ = 0 for a square pyramidal geometry, and τ = 1 for a trigonal–bipyramidal geometry).^21^ This coordination is completed by four oxygen atoms from mtif ligands occupying the equatorial plane, with Cu–O bond lengths ranging from 1.916 (6) to 1.993 (6) Å and aqua ligands in the axial positions. There are two types of polyhedral cages in **OGU-3**. The cage A is composed of 10 SBUs and 11 mtif ligands, while the cage B consists of seven SBUs connecting to five mtif ligands. The total potential solvent volume of **OGU-3** was found to be 11366.1 Å^3^, accounting for 68.0% of the total unit cell volume, as calculated by PLATON. **OGU-3** has a tetranodal 4-c network with the point symbol of {4·6^4^·8}_2_{4^2^·6^4^}­{6^4^·8^2^}_2_{6^6^}.^8^

**Figure 1:**
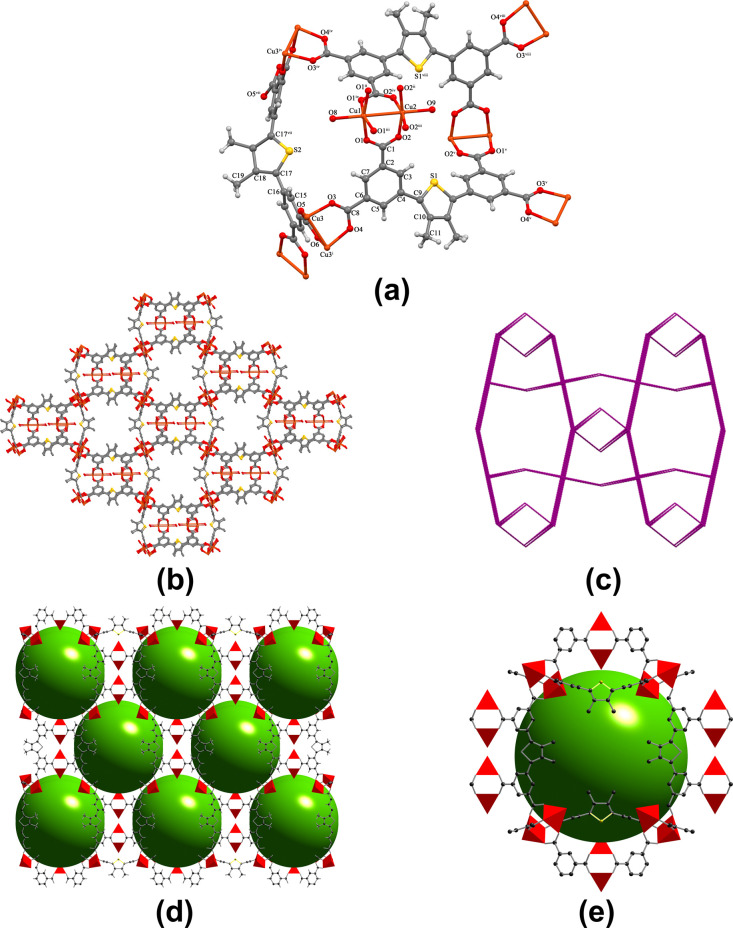
(a) Molecular structure of **OGU-3**,(b) three-dimensional (3D) framework of **OGU-3**, (c) simplified topological representation of the framework, (d, e) ball-and-stick models illustrating the pores present in the 3D structure.

**OGU-3** and **ZJNU-117** are structurally analogous three-dimensional porous MOFs built from dicopper paddlewheel units and diisophthalate-based linkers, crystallizing in the *Cmcm* space group and exhibiting the same tetranodal 4-connected topology. Their only structural difference is the presence of an additional −CH_3_ substituent in the linker of **OGU-3**, which causes minor steric effects that locally influence cage geometry without altering the underlying network connectivity.^8^

### 3.2 Powder X-ray Diffraction (PXRD) and Thermogravimetric Analysis

The phase purities of **ZJNU-117** and **OGU-3** were confirmed by PXRD. As shown in Figures S4 and S5, the experimental PXRD pattern matches well with that simulated from the single-crystal data, indicating its pure phase. Thermal analysis curves of the **OGU-3** were recorded in the temperature range of 303–1273 K under a dry air atmosphere to determine the thermal behavior of **OGU-3**. Additionally, to assess the framework stability in an inert environment, analysis was performed under a nitrogen (N_2_) atmosphere between 303 and 1023 K. A mass loss of approximately 41.75% was observed in a temperature range from 303 to 537 K from the TG curve of **OGU-3**, which corresponds to the removal of solvent molecules (calcd 41.10%) (Figure S6). Upon further heating, the decomposition of the organic ligand occurred in the range of 537–603 K, leaving CuO as the final decomposition residue. The identity of this residue was confirmed by PXRD analysis (Figure S7), which showed a pattern in excellent agreement with the standard diffraction data for copper­(II) oxide (Reference codes: ICSD 87122 and ICDD 98-008-7122).

During the synthesis of **OGU-3**, DMF and water molecules were incorporated into the framework as guest species. These molecules partially blocked the porous channels, limiting the material’s accessible surface area and potential adsorption capacity. To fully reveal the framework’s porosity and enhance its performance, activation of **OGU-3** was necessary. The activation process was carried out by soaking the as-synthesized **OGU-3** in acetone for 1 week, with the acetone being refreshed daily. Following this, the acetone-exchanged sample of **OGU-3** was degassed under high vacuum at 353 K for 12 h. The color of the **OGU-3a** changed from pale blue to deep purple-blue, indicating the exposure of unsaturated metal sites within the framework of **OGU-3**, as well as the removal of DMF and water molecules from the pores of the framework. The crystallinity and structural integrity of **OGU-3** after activation were assessed by PXRD. The PXRD pattern of the activated sample closely matched that of the as-synthesized material, confirming that the framework structure remained intact during the activation process. This result indicates that the removal of guest DMF and water molecules did not lead to framework collapse or significant structural rearrangement.

To further investigate the thermal stability of the framework, variable-temperature powder X-ray diffraction (VT-PXRD) measurements were performed in the temperature range of 298–573 K. As shown in Figure S8, the diffraction patterns remain essentially unchanged up to 523 K, indicating that the crystalline framework is preserved during the heating process. At higher temperatures, a gradual decrease in peak intensity is observed, suggesting the onset of structural degradation. These results are consistent with the thermogravimetric analysis and confirm that the framework maintains its structural integrity up to approximately 523 K.

Thermal analysis of the activated **OGU-3** was conducted to verify the complete removal of solvent molecules. The TG curve of the activated sample exhibited no significant weight loss below 523 K, further supporting the successful elimination of residual solvent molecules from the pores. These results confirm that the activation protocol yielded a solvent-free, open-framework version of **OGU-3** suitable for subsequent gas sorption studies (Figure S9). Furthermore, the structural stability of **OGU-3** under ambient conditions was investigated. The PXRD pattern of the sample after exposure to ambient air for 3 days remains in excellent agreement with the as-synthesized material, confirming that the framework maintains its crystallinity and structural integrity against moisture (Figure S5).

After establishing the crystalline phase and structural integrity by PXRD analysis, the permanent porosity of the framework was probed using N_2_ adsorption–desorption measurements at 77 K, consistent with the pore architecture revealed by the crystal structure. Prior to the measurements, the compound was activated through soaking in acetone (1 week) and heating at 353 K under vacuum. The N_2_ sorption isotherms of **OGU-3** exhibit a reversible type-I isotherm reflecting the nature of the micropores with permanent microporosity (Figure 2a). BET, in which the pressure ranges were determined according to the Rouquerol plots (Figure S10) and Langmuir surface areas of **OGU-3** calculated from N_2_ isotherm are 2612 and 2940 m^2^/g, respectively, which is comparable to the nonsubstitute MOF analogue reported in the literature.^8^ The N_2_ adsorption isotherm analyzed using the nonlocal density functional theory (NLDFT) model reveals micropore size distribution around 10.6 Å (Figure 2a, inset).

**Figure 2:**
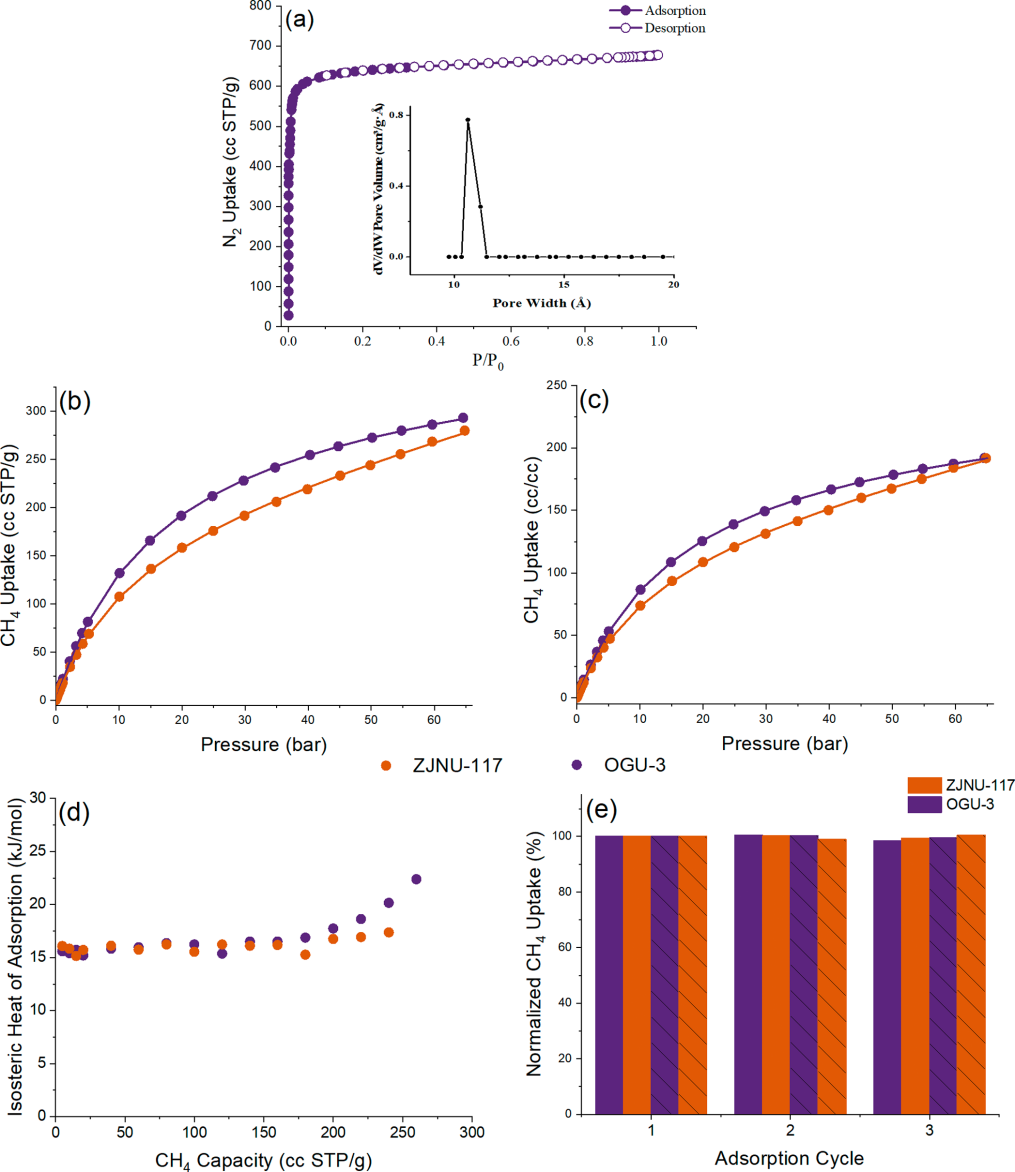
(a) N_2_ adsorption–desorption isotherms at 77 K for **OGU-3** (inset: pore-size distributions), volumetric CH_4_ adsorption behavior of **ZJNU-117** and **OGU-3** at 298 K and pressures up to 65 bar. Adsorption capacities are presented using (b) gravimetric (cc STP/g) and (c) volumetric (cc/cc) bases. Experimental data and fitted models are shown as symbols and lines, respectively. (d) Isosteric heat of adsorption of CH_4_ for **ZJNU-117** and **OGU-3**.(e) Cyclic regenerability of **ZJNU-117** and **OGU-3** for CH_4_ adsorption at 298 K. Solid bars correspond to adsorption at 35 bar CH_4_, while dashed bars correspond to adsorption at 65 bar.

### 3.3 Gas Adsorption Studies

Cu-MOFs with open metal sites have attracted significant interest in gas adsorption studies. Hence, CO_2_, CH_4_, and N_2_ adsorption/desorption isotherms were collected at various conditions. The corresponding uptakes of the **OGU-3** were measured as 144.7, 35.9, and 7.1 cc STP/g for CO_2_, CH_4_, and N_2_ at 273 K up to 1 bar. These results indicated the selective adsorption of CO_2_ over the other gases under these conditions. Moreover, CO_2_ adsorption–desorption isotherms were recorded at 298 K until 1.2 bar (Figure S11). The adsorption amount of **OGU-3** (69.7 cc STP/g) at 298 K and 1.0 bar is comparable to the reported unsubstituted compound.^8^ The slight decrease at the CO_2_ adsorption capacity could be due to methyl groups on the linker of **OGU-3**.

As copper-based MOFs have been extensively studied for CH_4_ storage at high pressure, we extended our analysis on CH_4_ uptake to a wider range of conditions. High-pressure CH_4_ adsorption isotherms were measured for **OGU-3** at 298, 303, and 308 K, spanning a pressure range of 0–65 bar, as shown in Figure 2b. For comparison, analogous data were also collected under identical measurement conditions for **ZJNU-117**, which possesses a similar structure to **OGU-3**, differing only by the absence of a methyl group substitution on the organic ligand.^8^ Experimentally measured adsorption isotherms were further converted to volumetric adsorption capacity, see Figure 2c, by using the crystallographic densities of desolvated structures by using Zeo++^22^ at as near ambient temperature as possible.^23^

**Figure 3:**
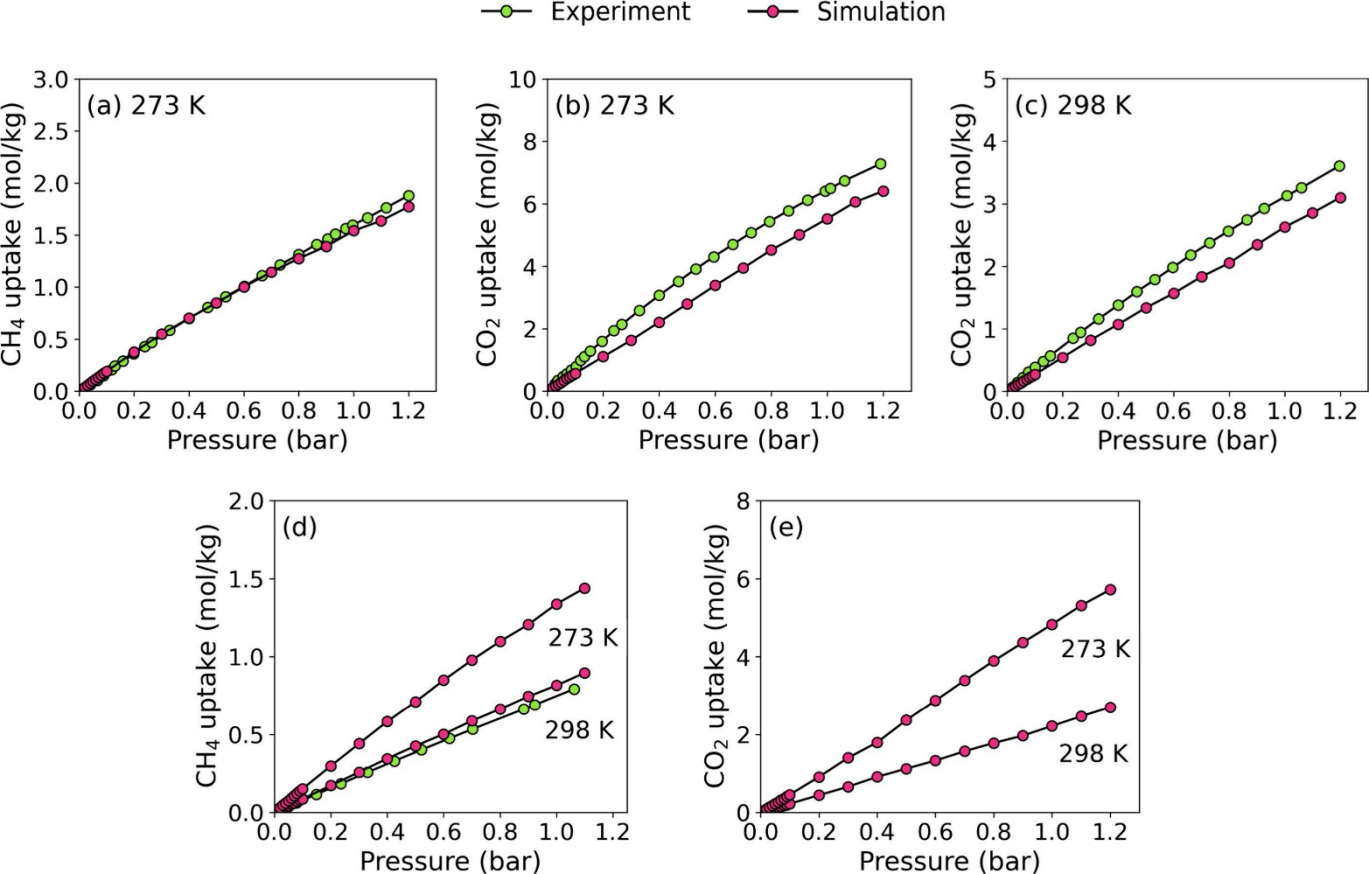
Comparison between simulated and experimental CH_4_ and CO_2_ uptakes in **OGU-3** over the pressure range of 0.01–1 bar at (a, b) 273 K and (c) 298 K. Comparison between simulated and experimental CH_4_ uptakes in **ZJNU-117** over the pressure range of 0.01–1 bar at (d) 273 and 298 K. (e) Simulated CO_2_ adsorption isotherms of **ZJNU-117** over the pressure range of 0.01–1 bar at 273 and 298 K.

The introduction of methyl (−CH_3_) groups into the organic linker of **ZJNU-117** to form **OGU-3** leads to a pronounced enhancement in methane adsorption capacity across the entire investigated pressure (0.1–65 bar) and temperature (298–308 K) ranges. This improvement is evident in both gravimetric and volumetric metrics, highlighting the effectiveness of nonpolar ligand functionalization for optimizing physisorptive CH_4_ storage as shown in Figure 2.

At all temperatures, **OGU-3** consistently outperforms the parent framework, with the most notable gains observed at moderate to high pressures. At 298 K and 65 bar, **OGU-3** exhibits a gravimetric uptake of 292.5 cc STP/g compared to 277.6 cc STP/g for **ZJNU-117**, see Figure 2b, corresponding to an enhancement of approximately 5.4%. A similar improvement is maintained at elevated temperatures, with **OGU-3** retaining 267.7 cc STP/g at 35 °C and 65 bar, compared to 246.5 cc STP/g for **ZJNU-117** at the same condition. Notably, the relative decrease in uptake upon heating from 298 to 308 K is smaller for **OGU-3**, indicating improved thermal robustness of methane adsorption following methyl functionalization.

Although **OGU-3** and **ZJNU-117** are crystallographically analogous and share the same tetranodal 4-connected topology, a noticeable difference is observed in their BET surface areas. **OGU-3** exhibits a BET surface area of approximately 2612 m^2^/g, which is slightly lower than that reported for **ZJNU-117** (2820 m^2^/g). This reduction may be attributed to the presence of the additional −CH_3_ substituent in the ligand of **OGU-3**. The methyl substituent introduces mild steric hindrance within the pore environment, slightly reducing accessible pore volume and surface area while subtly modifying cage geometry and aperture size. These structural changes may influence diffusion pathways and confinement effects, demonstrating that small variations in linker functionality can fine-tune the textural properties of isoreticular MOFs despite identical topology.

To further confirm this inference, *Q*_st_ values were determined by using the Clausius–Clapeyron equation and the corresponding van’t Hoff plots^25^ as shown in Figure S12. Data presented in Figure 2d demonstrated that the *Q*_st_ values for **OGU-3** ranges from approximately 15.2 to 22.4 kJ/mol over a CH_4_ uptake range of 5–260 cc STP/g, whereas **ZJNU-117** exhibits a narrower *Q*_st_ range of 15.1–17.4 kJ/mol over a comparable CH_4_ capacity, as illustrated in Figure 2d. Accordingly, at low methane uptakes (below 100 cc STP/g), both materials display relatively constant *Q*_st_ values characteristic of weak physisorptive interactions dominated by van der Waals forces.^26^ In this region, **OGU-3** shows similar *Q*_st_ values of around 16.0 kJ/mol, indicating that the initial adsorption sites in both frameworks possess similar energetic characteristics. This behavior suggests that methyl functionalization does not significantly alter the strongest primary adsorption sites but instead preserves a homogeneous energy landscape at low coverage. As the CH_4_ loading increases beyond 150 cc STP/g, distinct differences between the two materials become apparent. In **OGU-3**, the isosteric heat of adsorption increases progressively with capacity, reaching 18.6 kJ/mol at 220 cc STP/g and then rising sharply to 22.4 kJ/mol at the highest loading of 260 cc STP/g. In contrast, **ZJNU-117** exhibits a much more modest increase in *Q*_st_, remaining largely in the 15–17 kJ/mol range even at high loadings. Consequently, methyl functionalization enhances methane adsorption and leads to higher volumetric capacity at moderate pressures up to 55 bar. However, at higher pressures (∼65 bar), the volumetric methane capacities of **OGU-3** and **ZJNU-117** converge, which can be attributed to the larger pore size and pore volume of **ZJNU-117** resulting from the absence of methyl substituents, confirming the trend observed in *Q*_st_ results. Overall, the enhanced methane uptake of **OGU-3** is not due to stronger intrinsic framework–methane interactions, as the *Q*_st_ values for both materials are nearly identical at low loadings (∼16 kJ/mol) as shown in Figure 2d. Instead, the −CH_3_ groups slightly constrain the pore geometry, enhancing the confinement effect and promoting stronger adsorbate–adsorbate interactions as the framework approaches saturation.^27^ This geometric optimization rationalizes the improved methane storage performance of **OGU-3** with increasing coverage, illustrating how subtle linker modifications can fine-tune gas packing efficiency in isoreticular MOFs.^24^

Similarly, the fact that **OGU-3** exhibits higher volumetric uptake values (cc STP/cc) than **ZJNU-117** at all tested conditions confirms that the performance enhancement is not solely associated with the increased gravimetric capacity but also reflects more efficient packing of methane within the pore space. Consistently, at 298 K and 65 bar, the volumetric uptake increases from 190.4 cc STP/cc for **ZJNU-117** to 191.6 cc STP/cc for **OGU-3**, as shown in Figure 2b, demonstrating that methyl functionalization improves the storage efficiency of the adsorbate.

At 35 bar, **OGU-3** exhibits a notably high volumetric CH_4_ uptake of 158.9 cc (STP)/cc, positioning it among the most efficient methane adsorbents in its density range. Comparison presented in Table S3 shows that this value surpasses those of several high-surface-area but low-density frameworks, such as NU-111 (138 cc (STP)/cc), MOF-177 (126 cc (STP)/cc), and PCN-80 (147 cc (STP)/cc), demonstrating the advantage of **OGU-3**’s moderate framework. Compared to well-known high-performing MOFs like UTSA-76 (211 cc (STP)/cc), MAF-38 (226 cc (STP)/cc), and CuBTC (227 cc (STP)/cc), **OGU-3** displays slightly lower capacity but remains mostly competitive (Table 1). This relatively modest pressure response suggests that **OGU-3**’s pore network may consist predominantly of narrow-sized micropores with limited high-pressure accessibility. Nevertheless, the 35 bar performance highlights **OGU-3** as a promising candidate for moderate-pressure CH_4_ storage, offering an advantageous balance between framework density and adsorption efficiency.

**Table 1:** Literature Comparison of MOFs for CH_4_ Storage

				**Total Uptake** (cc STP/cc)				
	***S*_BET_** (m^2^/g)	**Pore Volume** (cm^3^/g)	**Density** (g/cm^3^)	35 bar	65 bar	**Temperature (K)**	***Q*_st_** (kJ/mol)	**Ref**
**OGU-3**	2612	0.954	0.655	158.9	191.6	298	15–22	this work
ZJNU-117	2820	1.115	0.686	142.2	190.4	298	15-17	this work
UTSA-76	2820	1.06	0.699	211	257	298	15.4	^29^
NOTT-102	3342	1.268	0.587	181	237	298	16.0	^6^
CuBTC	1850	0.78	0.883	227	267	298	17.0	^4^
MAF-38	2022	0.808	0.761	226	263	298	21.6	^2^
NOTT-101	2805	1.08	0.684	194	239	298	15.5	^6^
NOTT-103	2958	1.157	0.643	193	236	298	15.9	^6^
NU-125	3120	1.29	0.578	182	232	298	15.1	^4^
ZJU-35	2899	1.156	0.657	177	227	300		^30^
MOF-5		1.4	0.621	150	214	298	12.3	^31^
NU-111	4930	2.09	0.409	138	206	298	14.2	^4^
MOF-177	4500	1.89	0.43	126	193	298		^32^
MIL-101(Cr)	4230	2.15	0.44	130	198	303	18.0	^33^
MIL-100(Cr)	1900	1.1	0.7	144	202	303	19.0	^33^
NU-140	4300	1.97	0.426		200	298	14.0	^34^
UTSA-20	1620	0.66	0.909	184	230	298	18.2	^4^
PCN-80	3850	1.47	0.574	147	196	296		^35^
NU-1100	4020	1.53	0.467		180	298	13.7	^36^
PCN-14	2000	0.85	0.829	195	230	298	18.7	^4^

The cyclic stability of **OGU-3** and **ZJNU-117** was assessed at 25 °C by repeating CH_4_ adsorption measurements at 35 and 65 bar, with uptakes normalized to the first measurement. At 65 bar, **OGU-3** exhibits excellent reproducibility, with normalized uptakes of 100.3 and 99.7% in the second and third measurements, respectively, indicating negligible variation (±0.3%, less than the error range of measurements) upon cycling as shown in Figure 2e. In comparison, **ZJNU-117** shows slightly larger fluctuations, with normalized uptakes of 99.0 and 100.5%, reflecting a broader dispersion (but still within the error range of measurements) around the initial value. A similar trend is also observed at 35 bar. **OGU-3** retains 100.5 and 98.5% of its initial uptake in the second and third measurements, respectively, while **ZJNU-117** maintains 100.3 and 99.5%. Overall, both materials demonstrate good cyclic stability; however, the smaller deviation in normalized uptake for **OGU-3**, particularly at high pressure, confirms that methyl functionalization on the thiophene ring enhances the methane affinity without compromising adsorption reversibility or structural robustness.^28^

### 3.4 Molecular Simulation Studies

The simulation results for CO_2_ and CH_4_ adsorption in **OGU-3** show good agreement with the experimental data over the pressure range of 0.01–1 bar at 273 K, as illustrated in Figure 3. Figure 3a,b demonstrate that the simulated CO_2_ uptakes (0.06–5.52 mmol/g) are in good agreement with the experimental values (0.08–6.49 mmol/g). Similarly, the simulated CH_4_ uptakes (0.02–1.77 mmol/g) closely match the experimental measurements (0.016–1.75 mmol/g). A similar trend is also observed at 298 K, where the simulated CO_2_ uptakes (0.03–2.63 mmol/g) remain consistent with the experimental CO_2_ uptakes (0.03–3.13 mmol/g). Figure 3d compares the experimental and simulated CH_4_ adsorption of **ZJNU-117** at 298 K, also showing strong agreement. Notably, **OGU-3** exhibits higher CO_2_ and CH_4_ uptakes (2.63 and 0.95 mol/kg at 1 bar) than **ZJNU-117** (2.22 and 0.81 mmol/g at 1 bar) at 298 K, as shown in Figure 3d,e which can be attributed to the presence of methyl groups attached to the thiophene ring in **OGU-3** that provide a confinement effect and extra interaction sites for CH_4_ and CO_2_ molecules.

## 4 Conclusion

A new thiophene-functionalized Cu-based metal–organic framework, **OGU-3**, was successfully synthesized and thoroughly characterized. Structural analysis and gas sorption studies confirm that **OGU-3** possesses a robust three-dimensional microporous architecture with a high BET surface area exceeding 2612 m^2^/g. Gas adsorption studies show selective uptake of CO_2_ over CH_4_ and N_2_ at 273 K and 1.0 bar (144.7, 35.9, and 7.1 cc STP/g), respectively) and outstanding methane storage at 298 K and 65 bar of 292.5 cc STP/g (191.6 cc STP/cc). Cyclic measurements confirm excellent adsorption reversibility and structural stability, with smaller deviations compared to the nonmethyl-substituted analogue **ZJNU-117**, highlighting that methyl functionalization enhances methane affinity without compromising performance. These results demonstrate that heteroatom- and substituent-functionalized ligands effectively tune porosity, selectivity, and gas–framework interactions, providing valuable design principles for advanced MOFs targeting efficient gas storage and separation applications.

## Supplementary Material



## Notes

Synthetic route and NMR spectra of H_4_mtif; FT-IR and PXRD patterns; thermal analysis (TG/DTG/DTA); BET surface area analysis; gas adsorption isotherms; van’t Hoff plots; and crystallographic data and selected bond parameters for **OGU-3** (PDF)
