# Supplementary material for: Design and Synthesis of a High-Performance Copper(II) Metal–Organic Framework Featuring Large Surface Area and Enhanced Methane Adsorption Capacity from a Thiophene-Functionalized Diisophthalic Acid Ligand
Source: Inorg Chem. 2026 Mar 30;65(14):8094–103. doi: 10.1021/acs.inorgchem.6c00691 (PMC13081000; doi:10.1021/acs.inorgchem.6c00691)
Supplement: Supplementary file 1 [file ic6c00691_si_001.pdf]

Supporting Information

Design and Synthesis of a High-Performance Copper(II) Metal–Organic Framework Featuring Large Surface Area and Enhanced Methane Adsorption Capacity from a Thiophene-Functionalized Diisophthalic Acid Ligand

Okan Zafer Yeşilel[a,*], Ferihan Tataş Coşkun[a], Ahmet Safa Aydogdu[b,c], Alper Uzun[b,c,d], Hasan Can Gülbalkan[b], Seda Keskin[b,c], Kamuran Görgün[a], Mürsel Arıcı[a]

[a]Department of Chemistry, Eskişehir Osmangazi University, Faculty of Science, 26040 Eskişehir, Türkiye

[b]Department of Chemical and Biological Engineering, Koç University, Rumelifeneri Yolu, Sarıyer, 34450 Istanbul, Türkiye

[c]Koç University Hydrogen Technologies Center (KUHyTech), Koç University, Rumelifeneri Yolu, 34450 Sariyer, Istanbul, Türkiye

[d]Koç University Surface Science and Technology Center (KUYTAM), Koç University, Rumelifeneri Yolu, 34450 Sariyer, Istanbul, Türkiye

*Corresponding author: yesilel@ogu.edu.tr

Scheme S1. Synthetic route to H_4mtif

Fig. S1. ^{1}H NMR spectra (500 MHz) of H_4mtif in DMSO-d_6.

Fig. S2. ^{13}C NMR spectra (125 MHz) of H_4mtif in DMSO-d_6.

Fig. S3. The FT-IR spectrum of **OGU-3.**

Fig. S4. Comparison of experimental and simulated PXRD patterns for **ZJNU-117.**

Fig. S5. Comparison of the powder X-ray diffraction (PXRD) patterns for **OGU-3** in various states, including the experimental and simulated patterns of **OGU-3**, the activated framework (**OGU-3a**), and samples after gas adsorption and water treatment (**OGU-3@H_2O**).

Fig. S6. TG, DTG, and DTA curves of **OGU-3** recorded in air and the inset shows the TG curve obtained under an N_2 atmosphere.

Fig. S7. XRD patterns of the TG residue obtained from **OGU-3** and the CuO reference material (Reference codes: ICSD 87122 and ICDD 98-008-7122).

Fig. S8. Variable-temperature PXRD patterns of **OGU-3** collected in the temperature range of 298–573 K in air.

Fig. S9. Thermogravimetric analysis (TGA) curves of the as-synthesized **OGU-3** (black line) and the activated **OGU-3a** (blue line) samples under an air atmosphere.

Fig. S10. (a) Calculated Rouquerol plot for **OGU-3** along with the pressure ranges used for the BET surface area calculations (b) BET surface area plot of **OGU-3** obtained from N_2 isotherms at 77 K. The selected points are located in the pressure ranges of 0.008 to 0.04 according to the Rouquerol plots

Fig. S11. CO_2, CH_4, and N_2 adsorption-desorption isotherms up to 1.2 bar.

Fig. S12. Van't Hoff plot of ln K versus 1/T for the a) **ZJNU-117** and b) **OGU-3**, illustrating the temperature dependence of the adsorption equilibrium constant for CH_4. The slope of the linear fit corresponds to $-\Delta H/R$.

Table S1. Crystallographic data and refinement parameters for **OGU-3**

Table S2. Selected bond lengths (Å) and bond angles (°) for **OGU-3**

Table S3. CH_4 Adsorption capacity of **OGU-3** at temperatures of 298, 303, 308 K at given pressures of 35 and 65 bar.

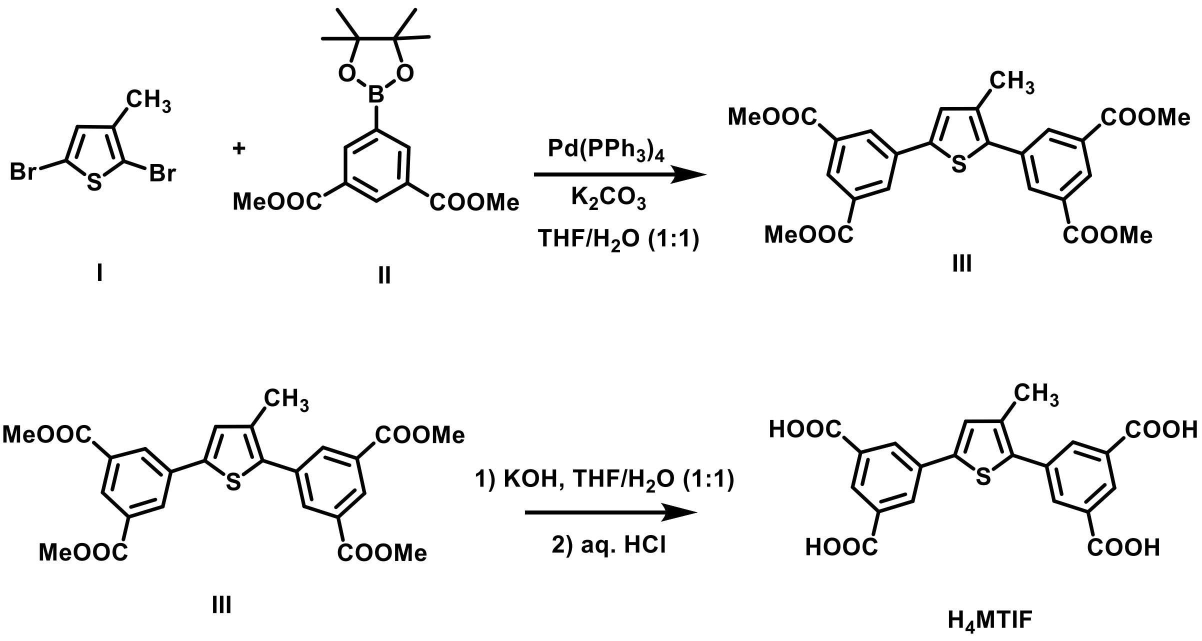

Scheme S1. Synthetic route to H_4mtif

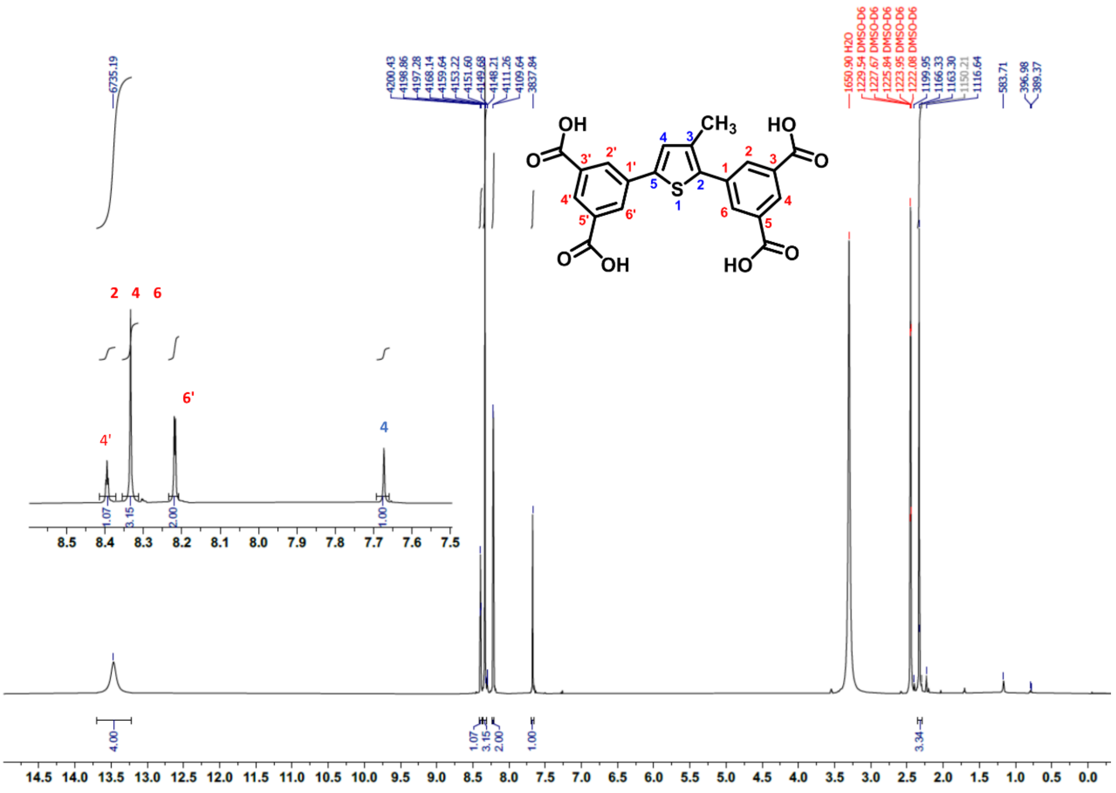

Fig. S1. ^{1}H NMR spectra (500 MHz) of H_4mtif in DMSO-d_6.

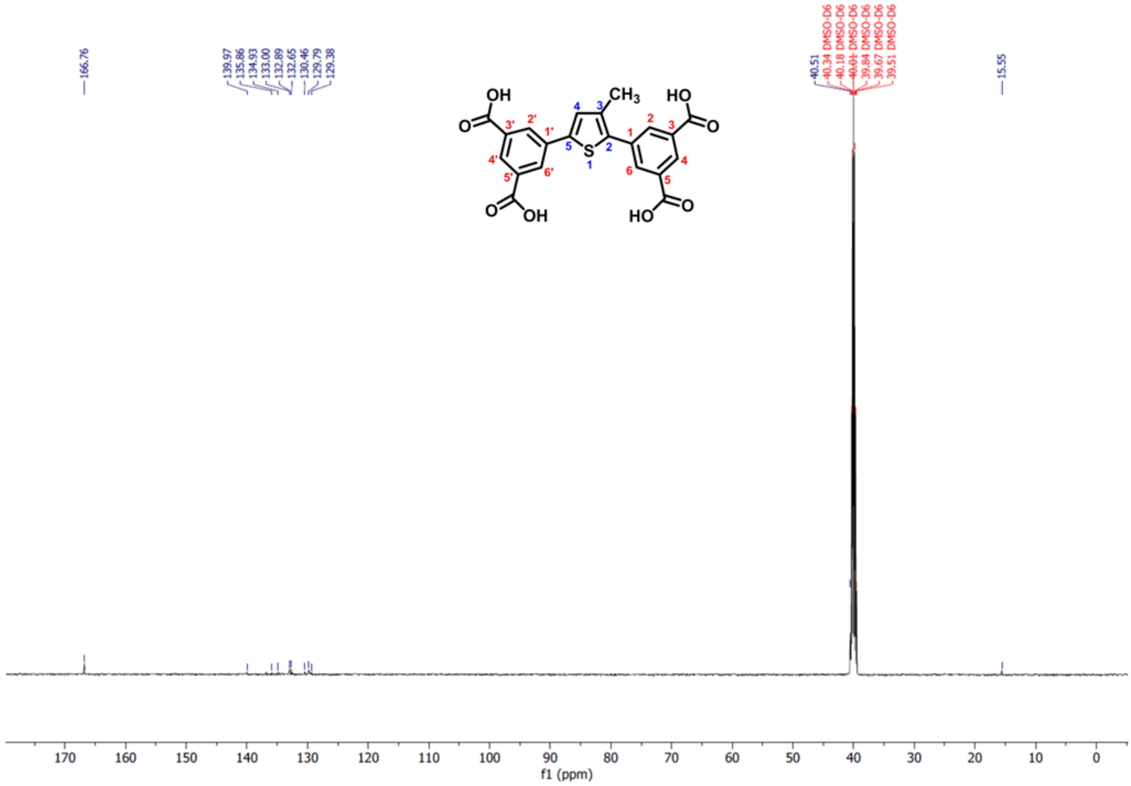

Fig. S2. ^{13}C NMR spectra (125 MHz) of H_4mtif in DMSO-d_6.

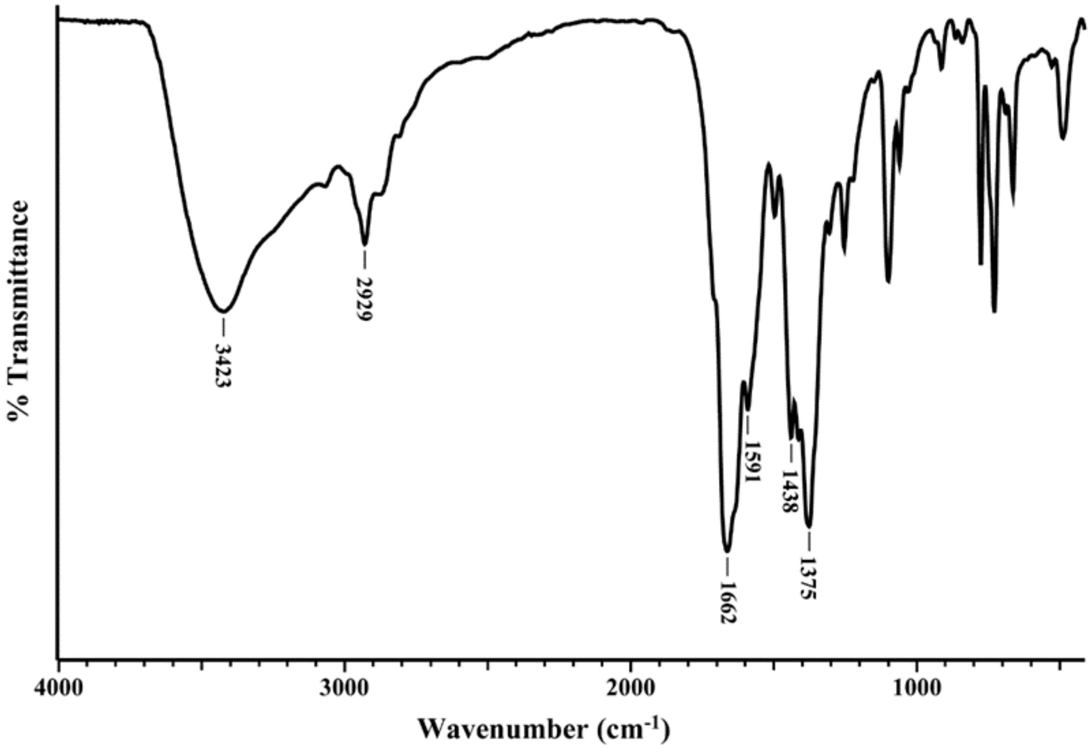

Fig. S3. The FT-IR spectrum of **OGU-3.**

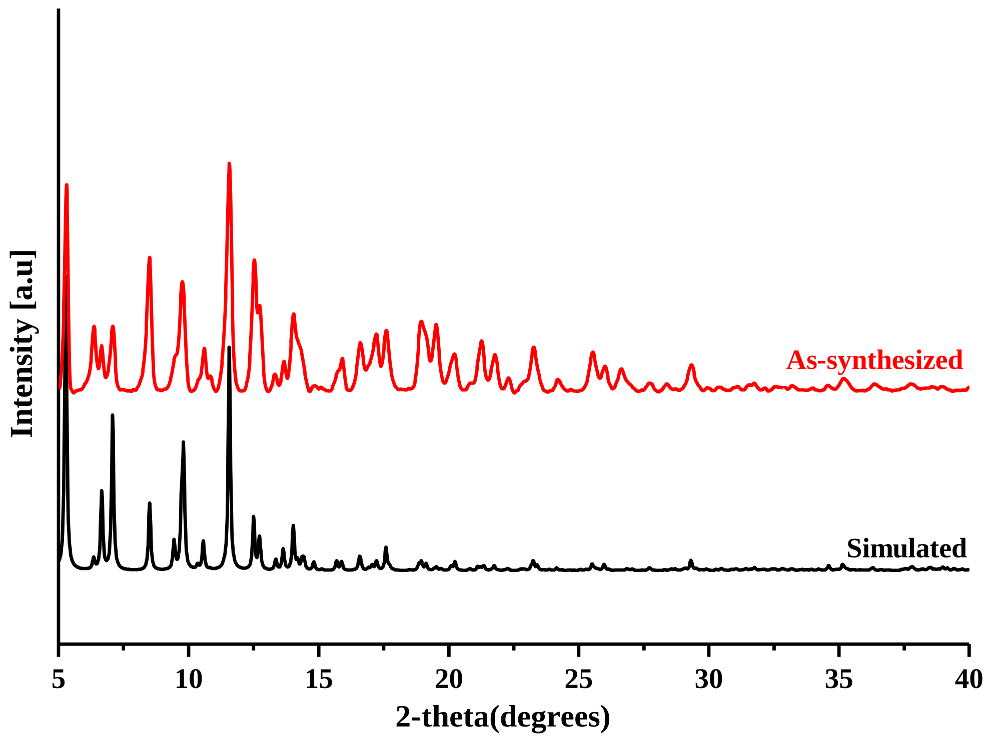

Fig. S4. Comparison of experimental and simulated PXRD patterns for **ZJNU-117.**

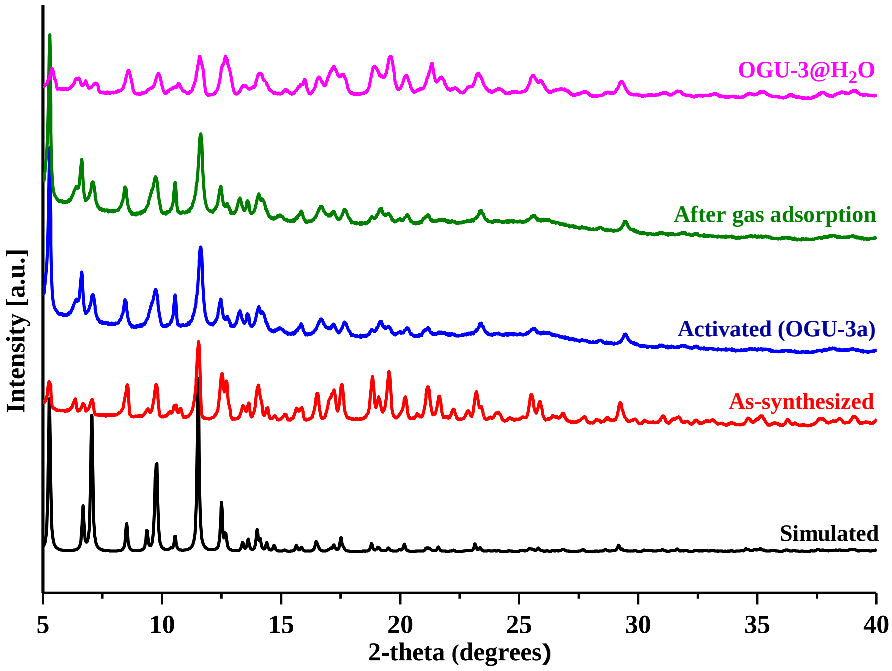

Fig. S5. Comparison of the powder X-ray diffraction (PXRD) patterns for **OGU-3** in various states, including the experimental and simulated patterns of **OGU-3**, the activated framework (**OGU-3a**), and samples after gas adsorption and water treatment (**OGU-3@H_2O**).

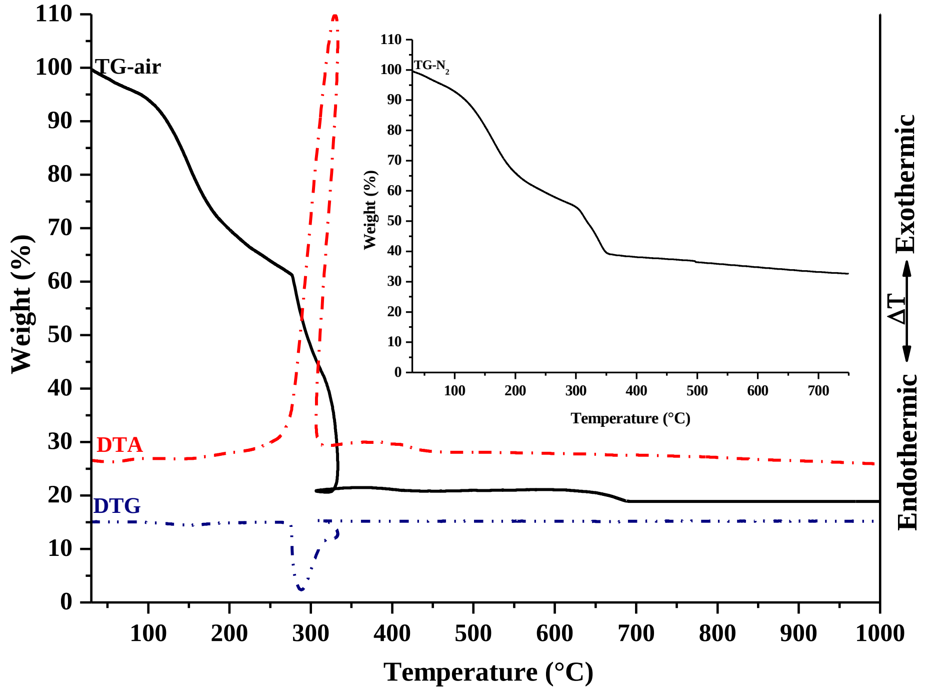

Fig. S6. TG, DTG, and DTA curves of **OGU-3** recorded in air and the inset shows the TG curve obtained under an N_2 atmosphere.

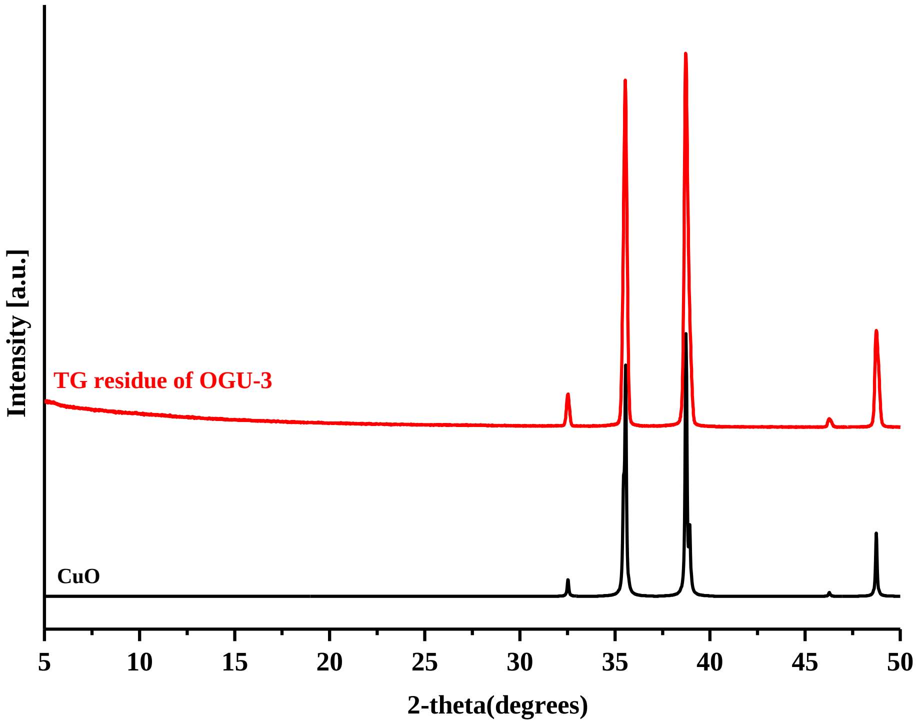

Fig. S7. XRD patterns of the TG residue obtained from **OGU-3** and the CuO reference material (Reference codes: ICSD 87122 and ICDD 98-008-7122).

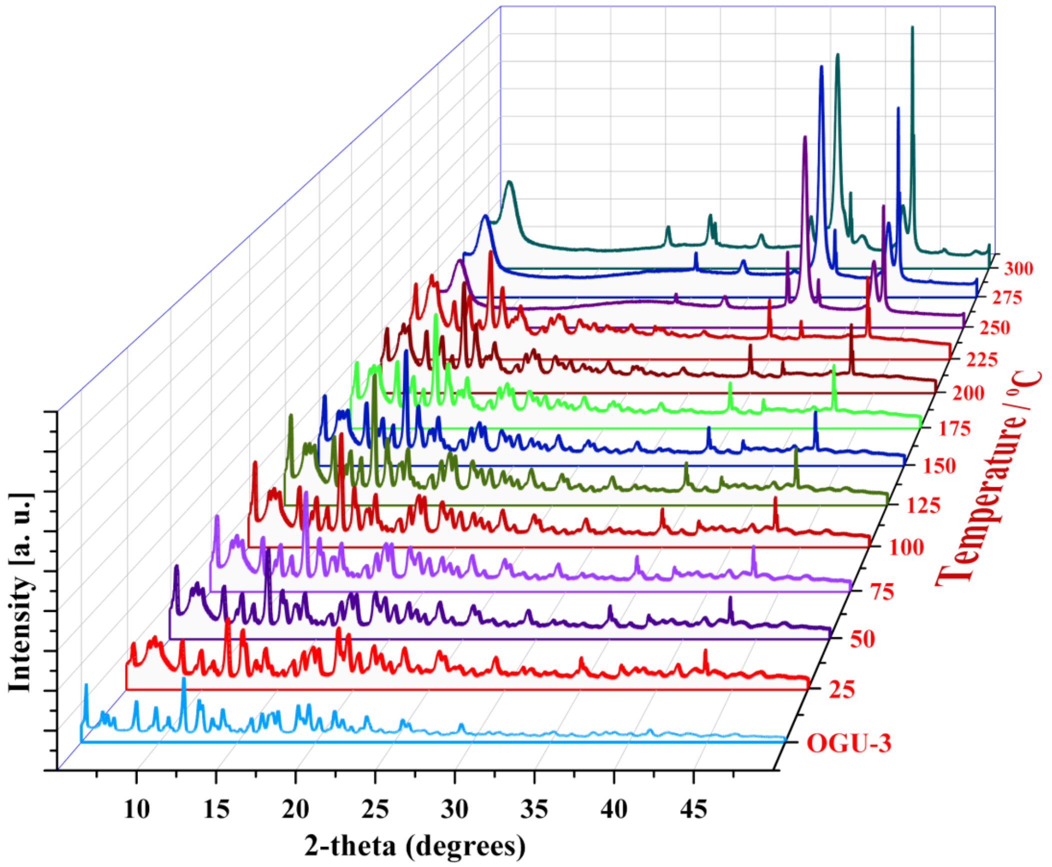

Fig. S8. Variable-temperature PXRD patterns of **OGU-3** collected in the temperature range of 298–573 K in air.

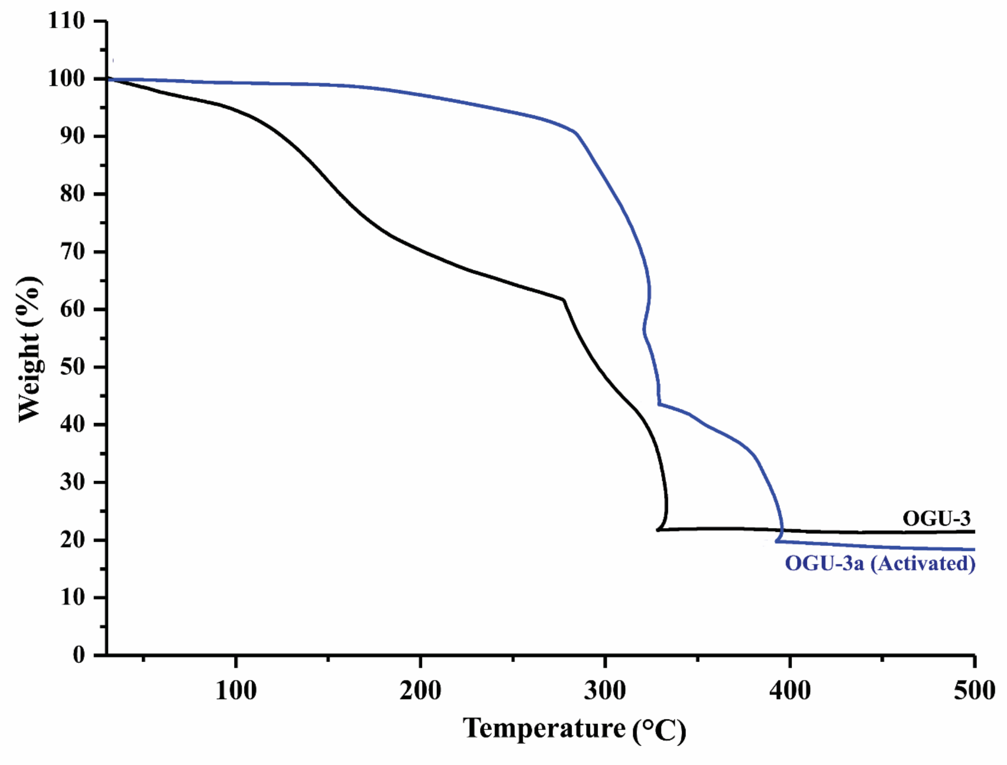

Fig. S9. Thermogravimetric analysis (TGA) curves of the as-synthesized **OGU-3** (black line) and the activated **OGU-3a** (blue line) samples under an air atmosphere.

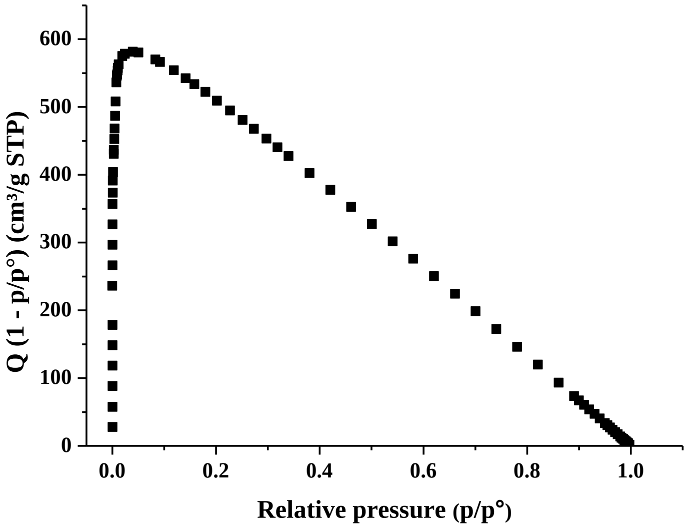

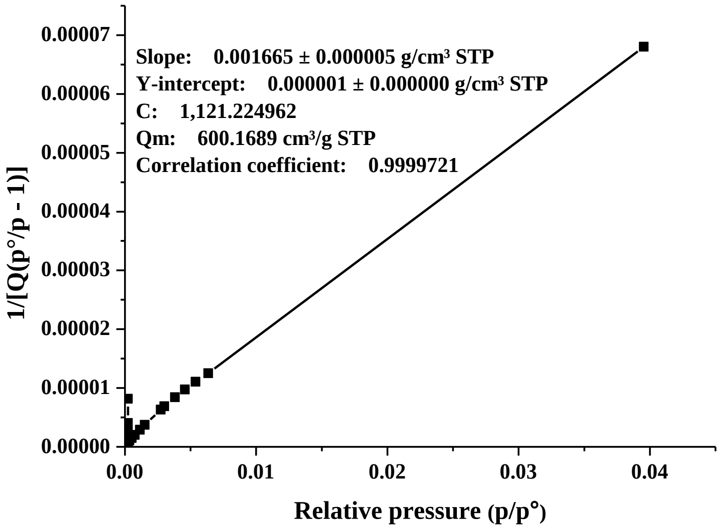

Fig. S10. (a) Calculated Rouquerol plot for **OGU-3** along with the pressure ranges used for the BET surface area calculations (b) BET surface area plot of **OGU-3** obtained from N_2 isotherms at 77 K. The selected points are located in the pressure ranges of 0.008 to 0.04 according to the Rouquerol plots

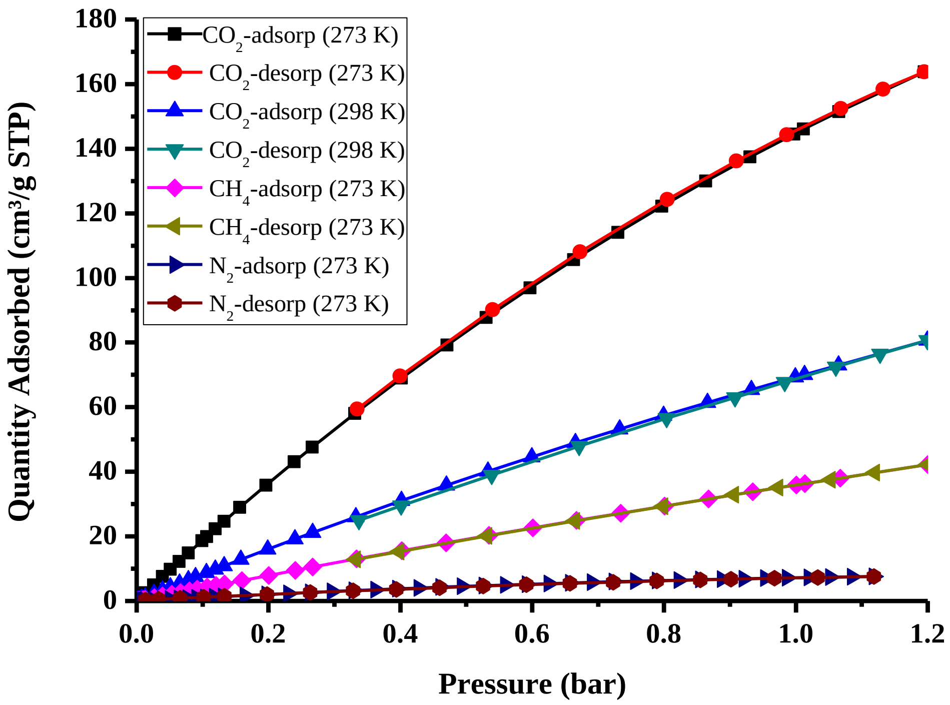

Fig. S11. CO_2, CH_4, and N_2 adsorption-desorption isotherms up to 1.2 bar.

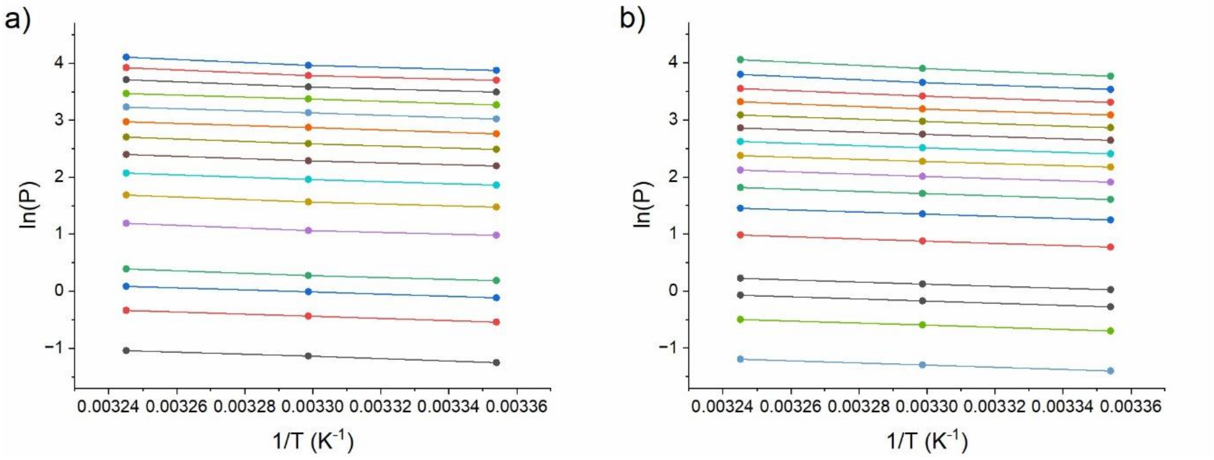

Fig. S12. Van't Hoff plot of ln K versus 1/T for the a) **ZJNU-117** and b) **OGU-3**, illustrating the temperature dependence of the adsorption equilibrium constant for CH_4. The slope of the linear fit corresponds to $-\Delta H/R$.

Table S1. Crystallographic data and refinement parameters for **OGU-3**

	**OGU-3**
**Empirical formula**	$C_{63}H_{30}Cu_6O_{30}S_3$
**Formula weight**	1744.29
**Diffractometer**	Bruker APEX II CCD
**Rad. /λ (Å)**	Mo-Kα/0.71073
**Temperature (K)**	273
**Crystal System**	Orthorhombic
**Space group**	Cmcm
**a (Å)**	26.431 (14)
**b (Å)**	33.516 (14)
**c (Å)**	18.867 (11)
**α (º)**	90
**β (º)**	90
**γ (º)**	90
**V ($Å^3$)**	16714 (15)
**Z**	4
**D_c (g cm^{-3})**	0.693
**μ (mm^{-1})**	0.82
$\theta_{max.}$ (º)	23.2
**Measured refls.**	12445
**Independent refls.**	4700
**R_{int}**	0.146
**S**	0.97
**R1/wR2**	0.073/0.212
$\Delta\rho_{max}/\Delta\rho_{min}$ **($eÅ^{-3}$)**	1.64/−1.88

Table S2. Selected bond lengths (Å) and bond angles (°) for **OGU-3**

**Bond distances**			
Cu1–O1	1.952 (6)	Cu3–O5	1.970 (6)
Cu1–O7	2.103 (12)	Cu3–O9	2.168 (6)
Cu2–O2	1.937 (6)	Cu3–O4^{i}	1.957 (5)
Cu2–O8	2.064 (11)	Cu3–O6^{i}	1.945 (6)
Cu3–O3	1.931 (5)		
**Bond Angles**			
O1^{ii}–Cu1–O1^{iii}	87.9 (4)	O2^{iii}–Cu2–O2	167.5 (3)
O1–Cu1–O1^{ii}	90.4 (4)	O2^{iv}–Cu2–O2^{iii}	90.1 (4)
O1–Cu1–O1^{iii}	165.9 (4)	O2^{ii}–Cu2–O2^{iii}	88.6 (4)
O1^{iv}–Cu1–O1^{iii}	90.4 (4)	O2^{ii}–Cu2–O2	90.1 (4)
O1^{iv}–Cu1–O1^{ii}	165.9 (4)	O2^{ii}–Cu2–O2^{iv}	167.5 (3)
O1–Cu1–O1^{iv}	87.9 (4)	O2^{iv}–Cu2–O2	88.6 (4)
O1–Cu1–O7	97.03 (18)	O2^{ii}–Cu2–O8	96.23 (17)
O1^{iii}–Cu1–O7	97.03 (18)	O2^{iii}–Cu2–O8	96.23 (17)
O1^{iv}–Cu1–O7	97.03 (18)	O2^{iv}–Cu2–O8	96.23 (17)
O1^{ii}–Cu1–O7	97.03 (18)	O2–Cu2–O8	96.23 (17)
O3–Cu3–O5	89.4 (3)	O4i–Cu3–O5	89.1 (3)
O3–Cu3–O4^{i}	168.1 (2)	O4^{i}–Cu3–O9	94.2 (3)
O3–Cu3–O6^{i}	88.9 (2)	O6^{i}–Cu3–O5	167.7 (2)
O3–Cu3–O9	97.7 (2)	O6^{i}–Cu3–O4^{i}	90.1 (2)
O5–Cu3–O9	93.5 (3)	O6^{i}–Cu3–O9	98.7 (3)
**Symmetry codes:** (i) $-x+3/2$, $-y+½$, $-z+1$; (ii) $-x+1$, y, z; (iii) $-x+1$, y, $-z+3/2$; (iv) x, y, $-z+3/2$.			

Table S3. CH_4 Adsorption capacity of **OGU-3** at temperatures of 298, 303, 308 K at given pressures of 35 and 65 bar.

		**ZJNU-117**		**OGU-3**	
**Temperature**	**Pressure (bar)**	**Uptake (cc STP/g)**	**Uptake (cc STP/cc)**	**Uptake (cc STP/g)**	**Uptake (cc STP/cc)**
298 K (25 °C)	35	207.3	142.2	242.6	158.9
	65	277.6	190.5	292.5	191.6
303 K (30 °C)	35	198.6	136.3	232.4	152.2
	65	265.3	182.0	280.3	183.6
308 K (35 °C)	35	187.0	128.3	221.3	144.9
	65	246.5	169.3	267.7	175.3
